# An *in vitro* evaluation of endodontic sealers and an antibiotic to assess their antimicrobial effect against *Enterococcus faecalis*

**DOI:** 10.2340/biid.v11.40646

**Published:** 2024-05-24

**Authors:** Elizabeth Madla-Cruz, Vanascheck Dasaev Villanueva-Pérez, Myriam A. De la Garza-Ramos, Jorge Jaime Flores-Treviño, Idalia Rodríguez-Delgado, Fanny López-Martinez

**Affiliations:** a Universidad Autónoma de Nuevo Leon, Facultad de Odontología, Calle Dr. Aguirre Pequeño and Silao, Colonia Mitras Centro, Monterrey, Nuevo Leon, Mexico CP 64460

**Keywords:** Amoxicillin, antimicrobial, endodontics, *Enterococcus faecalis*, direct contact test, root canal sealers

## Abstract

**Objective:**

This study aimed to compare the antimicrobial effect of three endodontic sealers (AH Plus, Mineral trioxide aggregate [MTA] Fillapex, and BioRoot RCS) with and without amoxicillin against *E. faecalis*.

**Methodology:**

Amoxicillin, equivalent to 10% of the sealers’ total weight, was mixed with the sealers. Another batch was prepared without amoxicillin. The direct contact test (DCT) and the agar diffusion test were used to assess the antibacterial effect. Results were analysed using one-way analysis of variance (ANOVA), the F-test, and the Kruskal-Wallis test.

**Results:**

AH Plus significantly suppressed *E. faecalis* without the addition of amoxicillin in the DCT (p = 0.011), while in the agar diffusion test, BioRoot RCS had a larger inhibition zone than the control (p < 0.001). When amoxicillin was added to the sealers, AH Plus (p = 0.003) and MTA Fillapex (p = 0.042) reduced *E. faecalis* growth. In contrast, all three sealers showed larger inhibition zones than the control (p = 0.001), with AH Plus displaying a larger inhibition zone than MTA Fillapex (p = 0.042) and BioRoot RCS (p = 0.032).

**Conclusions:**

It was thus concluded that the addition of amoxicillin to endodontic sealers enhances their antimicrobial activity against *E. faecalis*.

## Introduction

Microbes and their products are the main etiologic agents in pulpitis and apical periodontitis [1, 2]. Microorganisms are present after the chemo-mechanical preparation of the root canal [3, 4]. *Enterococcus faecalis* is associated with different forms of periradicular disease, including endodontic treatment failure and persistent infections [5]. The frequency of *E. faecalis* in persistent periradicular lesions is high. Failed root canal cases are nine times more likely to contain this microorganism [6]. Studies investigating the prevalence of infection in root-filled teeth found that apical periodontitis varied from 7% to 86% and post-treatment apical periodontitis from 10% to 62% [7]. Preventing microbial contamination of the root canal system and eliminating microorganisms from the infected root canal are the goals of endodontic treatment. Chemical-mechanical disinfection significantly reduces microorganisms in the infected root canal system; however, there are areas of the root canal where it is impossible to completely eliminate microorganisms [8]. Irrigation methods promote the activation of the irrigant towards areas outside the reach of instrumentation. If we add endodontic materials such as sealants, cements, pastes, putties and filling materials with antimicrobial activity, it will further reduce residual microorganisms and prevent possible reinfection [9]. For this reason, sealers with antibacterial properties may be useful in decreasing or avoiding microorganism growth [10].

Bio-ceramics are inorganic, non-metallic, biocompatible materials used in direct contact with living tissues in the medical and dental fields. Since they are chemically stable, non-corrosive, and interact well with organic tissues, more bio-ceramic materials have been developed and successfully used in endodontic treatments, including pulp capping, obturation, apical barrier formation, perforation repairs, and root-end filling [11, 12]. Their antimicrobial and antibiofilm properties are exerted during the setting process by increasing pH and ion release from the material [13]. The success of endodontics can be improved with the use of sealants with excellent sealing ability and antimicrobial properties [14].

AH Plus (Dentsply Sirona, Bensheim, Germany) is an epoxy resin-based cement that contains calcium tungstate and zirconium oxide. It has low solubility and disintegration, and good adhesion [15, 16]. Mineral trioxide aggregate (MTA) Fillapex (Angelus, Londrina, Brazil) is supplied with a base and catalyst paste. It is mixed in a syringe for application. Its composition after mixing is MTA, salicylate resin, natural resin, bismuth, and silica [17], which produce a colloidal gel that solidifies [18]. BioRoot RCS (Septodont, Saint-Maur-des-Fossés, France), a tricalcium silicate-based root canal sealer, is composed of tricalcium silicate, zirconium dioxide, and a water-based liquid with calcium chloride and a water-soluble polymer. This root canal sealer has fewer toxic effects on human periodontal ligament cells compared to other sealers, and induces a higher secretion of angiogenic and osteogenic growth factors [19].

Several studies have shown that anaerobic bacteria are associated with persistent root canal infections [20, 21]. These bacteria can survive in a necrotic environment lacking blood and oxygen [22]. This condition makes root canal sealers with antibacterial characteristics imperative in endodontic treatment. Combining sealers with antibiotics could potentiate their antimicrobial effect, and reduce the critical concentration of microbes necessary for a favourable host response [21]. Chronic alveolar infections are associated with pulpless teeth and lesions with no blood supply reaching the pulp space. A systemic antibiotic concentration that reaches the root canal is negligible because of this lack of circulation [22].

Some studies have evaluated the antimicrobial effects of amoxicillin, vancomycin, erythromycin, benzylpenicillin, and doxycycline against *E. faecalis* [23]. When used locally, a higher concentration of the drug is available [24]. Hoelscher et al. [21] demonstrated that sealant-antibiotic combinations containing amoxicillin, penicillin, clindamycin, and doxycycline could significantly increase growth inhibition zones compared to sealants alone. Likewise, Baer and Maki [20] reported that sealants mixed with amoxicillin had a greater inhibitory effect on *E. faecalis* growth than those without amoxicillin.

This study aimed to determine if a combination of a sealant and amoxicillin can inhibit *E. faecalis* growth and increase the antimicrobial effect of the sealant.

## Material and methods

### Ethics

The study protocol was reviewed and approved by the Ethics Committee of the School of Dentistry of the Universidad Autónoma de Nuevo León with registration no. SPSI-010613-00290.

### Materials and microorganisms

AH Plus (Dentsply Sirona, Bensheim, Germany), MTA Fillapex (Angelus, Londrina, Brazil), and BioRoot RCS (Septodont, Saint-Maur-des-Fossés, France) were mixed according to the manufacturer’s instructions. In a second preparation, sealers were weighed and mixed with crushed amoxicillin (Sigma-Aldrich Saint Louis, MO, USA), equivalent to 10% of the sealer’s total weight [21], and then prepared as before.

*Enterococcus faecalis* (ATCC 11420) was obtained from a microbial culture of the Center for Research and Development in Health Sciences. They were grown and maintained on brain-heart infusion (BHI) broth (Difco Laboratories, Detroit, MI) for 24 h at 37ºC in an anaerobic chamber. Broths were prepared in the laboratory according to the manufacturer’s specifications. The cells were harvested by centrifugation and resuspended in fresh medium. Sealers without the bacterial inoculum were the negative control, and the bacterial inoculum without sealers was the positive control.

### Direct contact test

The direct contact test (DCT) [25] was performed on 96-well microtiter plates (Nunc A/S, Thermo Fisher Scientific Inc., Copenhagen, Denmark). The sealers were placed on the side wall of each well in a vertical position. Thereafter, a bacterial suspension of 10^6^ cells (10 μl) was placed over the sealers. Finally, 245 μl of BHI broth was added to each well. Growth kinetics were measured with a microplate spectrophotometer with absorbance at 595 nm with readings every 30 min.

### Agar diffusion test

A 200 μl bacterial suspension with 2 × 10^6^ cells was seeded on agar plates. A 5-mm vertical well was drilled in the agar and filled with each sealer sample. Zinc oxide eugenol (ZOE) was used as the control because of its antibacterial effect. The plates were incubated at 37°C for 24 h, and bacterial growth was determined by measuring the inhibition zone.

Five parallel tests were performed in all experiments. Repetitions were performed for each experiment, and the result of the set material was evaluated after 24 h.

### Statistical analysis

The non-parametric Kruskal-Wallis test, one-way analysis of variance (ANOVA), and F-test were used. The statistical analysis was performed with Statistical Package for the Social Sciences (SPSS) v. 21. A *p*-value ≤ 0.05 was considered significant.

## Results

### Direct contact test

**Figure 1:**
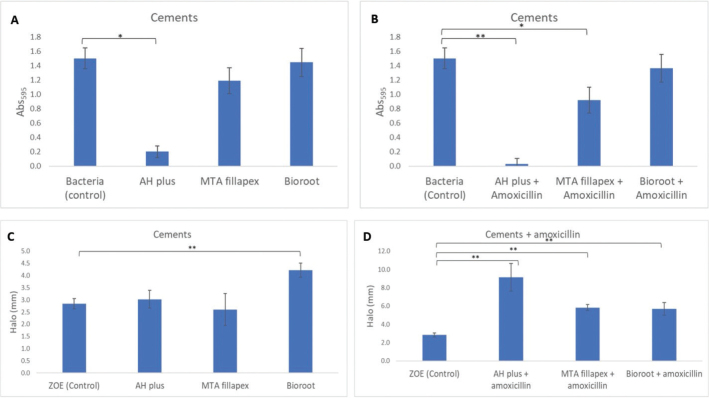
Mean absorbance of bacterial growth with the direct contact test in three sealers without (A) and with amoxicillin (B). The mean bacterial inhibition zone with the agar diffusion test in sealers without (C) and with amoxicillin (D). *The mean difference is significant at the p < 0.05 level; **The mean difference is significant at the p < 0.001 level. Note: the values on the Y scale in the lower figure differ between C and D.

The results of the DCT are shown in Figure 1. AH Plus without amoxicillin significantly reduced *E. faecalis* growth (*p* = 0.011) (Figure 1A). The antimicrobial effect of AH Plus was greater with amoxicillin (*p* = 0.003). MTA Fillapex (*p* = 0.042) also had a greater effect on *E. faecalis* growth with amoxicillin (Figure 1B). The antimicrobial effect of BioRoot RCS varied little with and without amoxicillin.

The upper section of Table 1 shows a comparison of the three antibiotic-free sealants against *E. faecalis*. It is observed that the AH Plus cement was one of those that had a significant effect compared to the control (*p* ≤ 0.011), together with BioRoot RCS (*p* ≤ 0.028).

**Table 1:** Comparison of sealers without and with amoxicillin in the direct contact test.

Sealer w/o amoxicillin	*t*−statistic	SD	*t*−statistic D	Sig.	Adj. Signif.
AH plus–MTA Fillapex	−4.000	3.873	−1.033	0.302	1.000
AH plus–BioRoot RCS	−8.500	3.873	−2.195	*0.028	0.169
AH plus–Bacteria (control)	−9.500	3.742	−2.539	*0.011	0.067
MTA Fillapex–BioRoot RCS	−4.500	3.162	−1.423	0.155	0.928
MTA Fillapex–Bacteria (control)	−5.500	3.000	−1.833	0.067	0.401
BioRoot RCS–Bacteria (control)	−1.000	3.000	−0.333	0.739	1.000
**Sealers with amoxicillin**					
AH plus + amoxicillin–MTA Fillapex + amox	−3.000	3.416	−0.878	0.380	1.000
AH plus + amoxicillin–BioRoot RCS + amox	−7.333	3.416	−2.147	*0.032	0.191
AH plus + amox–Bacteria (control)	−9.200	3.005	−3.011	*0.003	0.016
MTA Fillapex +amox–BioRoot RCS + amox	−4.333	3.416	−1.269	0.205	1.000
MTA Fillapex + amox–Bacteria (control)	−6.200	3.055	−2.029	*0.042	0.254
BioRoot RCS + amox–Bacteria (control)	−1.867	3.055	−0.611	0.541	1.000

DCT: direct contact test; MTA: Mineral trioxide aggregate; amox, amoxicillin; w/o: without; *t*-statistic: hypothesis test statistic; SE: standard error; *t*−statistic D: hypothesis test statistic deviation; Sig.: significance; Adj. Signif.: adjusted significance.

* Significant result.

When the cements were mixed with amoxicillin (Table 1, lower segment), there was a significant difference in the presence of *E. faecalis*. AH Plus with amoxicillin showed the best results (*p* = 0.003) together with BioRoot RCS (*p* = 0.032). MTA Fillapex was significantly different (*p* = 0.042) compared to BioRoot RCS with amoxicillin (*p* = 0.541) and MTA Fillapex with amoxicillin + BioRoot RCS with amoxicillin (NS).

### Agar diffusion test

**Figure 2:**
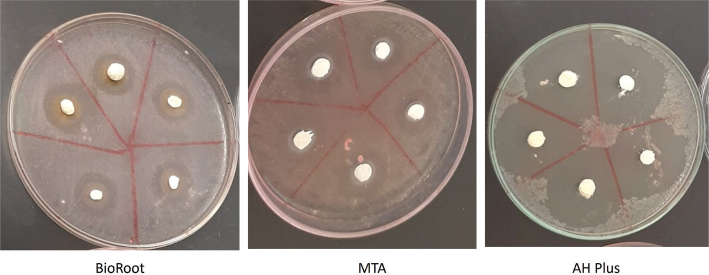
Results of the agar diffusion test of the three sealers with amoxicillin. MTA: Mineral trioxide aggregate.

The results of the agar diffusion test are shown in Table 2 and Figures 1C, D and Figure 2. BioRoot RCS without amoxicillin had the largest mean inhibition zone (*p* < 0.001), followed by AH Plus, and MTA Fillapex (Figure 1C). The sealers mixed with amoxicillin had larger mean inhibition zones: AH Plus was 9.1 mm (*p* = 0.001), MTA Fillapex, 5.8 mm (*p* = 0.042), and BioRoot RCS, 5.7 mm (*p =* 0.032) (Figure 1D). All three sealers with amoxicillin were more effective than the control (bacteria alone). However, only AH Plus and BioRoot RCS without amoxicillin were more effective than the control, but only BioRoot RCS was statistically significant.

**Table 2:** Agar diffusion test results without and with amoxicillin.

Sealer	Zone (mm)	*p*	Sealer + amoxicillin	Zone (mm)	*p*
ZOE (Control)	2.840 ± 0.207		ZOE (Control)	2.840 ± 0.207	
AH Plus	3.025 ± 0.359	0.808	AH Plus + amoxicillin	9.120 ± 1.509	0.001
MTA Fillapex	2.600 ± 0.656	0.720	MTA Fillapex + amoxicillin	5.825 ± 0.320	0.042
BioRoot RCS	4.220 ± 0.295	< 0.001	BioRoot RCS + amoxicillin	5.700 ± 0.698	0.032

Values are mean ± SD.

ZOE: Zinc oxide eugenol; MTA: Mineral trioxide aggregate.

*P-values* correspond to the comparison between the control and each treatment.

## Discussion

Microorganisms can persist after root canal preparation and reinfect the root canal. Systemic antibiotics in this scenario are unlikely to be beneficial [26]. We studied the antimicrobial efficacy of three sealers, AH Plus, MTA Fillapex, and BioRoot RCS, alone and after adding amoxicillin against *E. faecalis*. We found that an endodontic root canal sealer combined with an antibiotic reduced bacterial growth. Amoxicillin was used because it has a good antibiotic spectrum and is the first choice for endodontic infections [22, 27].

BioRoot RCS and AH Plus without amoxicillin showed greater antimicrobial efficacy than the control in the DCT. The antimicrobial effect of the three sealers in the DCT increased after adding amoxicillin. This increase was greatest with AH Plus. The effect of MTA Fillapex and BioRoot RCS in the agar diffusion test was similar. The differences in the findings could be due to variances in the two tests. In the disc diffusion test some bacteria may grow poorly or not at all, and the minimum inhibitory concentration cannot be determined. The DCT is more reliable because it directly evaluates the bactericidal effects of the sealer with and without the antibiotic. It is also more suitable to assay solid surfaces, and is independent of the diffusion properties of the tested material [25, 28].

We previously studied the antibacterial effect of three endodontic sealers, AH Plus, BioRoot RCS, and EndoSequence, plus ZOE, using the agar diffusion test and the DCT with the same *E. faecalis* strain. BioRoot RCS had an antimicrobial effect statistically similar to the ZOE control. EndoSequence and AH Plus had a lower antimicrobial effect than the control [29]. In contrast to our results, BioRoot RCS and EndoSequence had the largest inhibition zones. In the DCT, EndoSequence and AH Plus had a lower antimicrobial effect than the control.

The antimicrobial effect of AH Plus found in our study agrees with the findings of Kapralos et al. [30] who reported that AH Plus had high antibacterial activity using planktonic cells bacteria and bacteria in biofilms. Huang et al. [31] tested the antimicrobial activities of four endodontic sealers (GuttaFlow2, AH Plus, ProRoot MTA Fillapex, and RealSeal) with the agar diffusion test and the DCT. In their research, freshly mixed AH Plus had a strong antimicrobial effect, corroborating our results with the DCT. Kangarlou et al. [32] showed that AH Plus alone or combined with nanosilver did not show an antimicrobial effect against *E. faecalis* in samples after 1, 3, and 7 days. However, freshly mixed AH Plus without antibiotics showed antimicrobial activity against *E. faecalis*, corroborating our finding on set AH Plus. Finally, Baer and Maki [20] found that sealers without amoxicillin allowed growth similar to the positive control, similar to our findings with regard to AH Plus and MTA Fillapex, but not BioRoot RCS. This discrepancy can be because they used fresh sealers, and sealers set 1 day, 3 days, and 7 days after mixing. The three sealers with amoxicillin maintained their antimicrobial effect after 7 days.

*E. faecalis* is a common pathogen in root-filled teeth with periapical lesions and is susceptible to amoxicillin [21, 22]. Pinheiro et al. [23] found that *E. faecalis* isolates were susceptible *in vitro* to amoxicillin, amoxicillin-clavulanic acid, vancomycin, and moxifloxacin. Endodontic sealers with antibiotics have a peak antimicrobial activity at an antibiotic concentration of 10%. Increasing the concentration to 50% did not increase inhibition [21]. Accordingly, we chose to use an antibiotic concentration of 10% in this study; indeed, this concentration provided the sealers with an antimicrobial effect.

Although the antimicrobial effect of endodontic sealants has been widely investigated in the literature [33], and their effect against microorganisms has been proven in studies with various sealing cements combined with an antibiotic [20, 21], sealing cements are not marketed with an antibiotic included. This research supports the advantages of adding an antibiotic. Future research should consider modifications in the chemical properties of sealants when adding antibiotics.

Our study is not without limitations. It is an *in vitro* test performed in culture. Periapical infections are multi-species, and we used only *E. faecalis* and one antibiotic. The sealers were analysed 24 hours after setting in contrast to other studies in which fresh and different setting times were analysed.

In conclusion, an endodontic sealer combined with amoxicillin can inhibit *E. faecalis* growth, and possibly prevent root canal reinfection.

## Data Availability

The datasets generated and analyzed during the current study are available from the corresponding author upon reasonable request.

## Key messages

Endodontic sealers are used to avoid recurrent infection.

Combining endodontic sealers and antibiotics enhances their antibacterial activity.

This combination showed antimicrobial activity against *Enterococcus faecalis*.
